# Neuron-Specific Gene 2 (NSG2) Encodes an AMPA Receptor Interacting Protein That Modulates Excitatory Neurotransmission

**DOI:** 10.1523/ENEURO.0292-18.2018

**Published:** 2019-01-24

**Authors:** Praveen Chander, Matthew J. Kennedy, Bettina Winckler, Jason P. Weick

**Affiliations:** 1 Department of Neurosciences, University of New Mexico-Health Science Center, Albuquerque, NM 87131; 2 Department of Pharmacology, University of Colorado-Denver, Aurora, CO 80045; 3 Department of Cell Biology, University of Virginia, Charlottesville, VA 22908

**Keywords:** AMPAR, endosome, HMP19, human, synaptogenesis, trafficking

## Abstract

Neurons have evolved a number of unique protein-coding genes that regulate trafficking of protein complexes within small organelles throughout dendrites and axons. Neuron-specific gene 2 (NSG2) encodes for one of the most abundant proteins in the nervous system during perinatal development. NSG2 belongs to a family of small neuronal endosomal proteins but its function has remained uncharacterized to date. Here, we show that NSG2 is found in discrete punctae restricted to the somatodendritic arbors of developing mouse and human neurons, and a significant proportion of NSG2 punctae colocalize with postsynaptic HOMER1 and surface-expressed AMPA-type glutamate receptors (AMPARs) at excitatory synapses. Immunoprecipitation revealed that NSG2 physically interacts with both the GluA1 and GluA2 AMPAR subunits in mouse brain. Knock-out of NSG2 in mouse hippocampal neurons selectively impaired the frequency of miniature EPSCs (mEPSCs) and caused alterations in PSD95 expression at postsynaptic densities (PSDs). In contrast, NSG2 overexpression caused a significant increase in the amplitude of mEPSCs as well as GluA2 surface expression. Thus, NSG2 functions as an AMPAR-binding protein that is required for normal synapse formation and/or maintenance, and has unique functions compared with other NSG family members.

## Significance Statement

Because of their morphologic and functional complexity, neurons have evolved specialized proteins like those of the neuron-specific gene family [neuron-specific gene (NSG)1–NSG3]. Important developmental and synaptic functions have been attributed to NSG1/NEEP21 and NSG3/Calcyon, while the function of NSG2/HMP19/NVTA2 has remained uncharacterized. Here, we show that NSG2 localizes to a large proportion of excitatory synapses, interacts with AMPA-type glutamate receptors (AMPARs), and modulates their surface expression during synaptogenesis. Alterations of NSG2 expression affected both the amplitude and frequency of excitatory neurotransmission that is not compensated for by other NSG family members. Thus, NSG2 appears critical for excitatory synapse formation and/or maintenance and forced expression can promote increased synaptic efficacy.

## Introduction

Neuron-specific gene 2 (NSG2, neuronal vesicle trafficking-associated 2) belongs to the “neuron-specific gene” family of small, single-pass transmembrane proteins that localize to vesicular compartments within neuronal dendrites. The other two NSG family members are NSG1/NEEP21 and NSG3/Calcyon (Muthusamy et al., 2009; Norstrom et al., 2010; Lasiecka et al., 2014), which have been shown to have a number of critical protein trafficking functions primarily involved with neural development and synaptic plasticity. Interestingly, Human NSG2 shares only 50% sequence homology with NSG1 and a 30% sequence homology with NSG3 at the amino acid level (Muthusamy et al., 2009). NSG2 is one of the most highly expressed transcripts and proteins during human neural differentiation and synapse formation (Kang et al., 2011; Miller et al., 2014; Barford et al., 2017; Murillo et al., 2017). It is expressed widely throughout the brain (Sabéran-Djoneidi et al., 1995) with significant overlapping expression with NSG1 but some degree of divergence with NSG1 (Barford et al., 2017). At the cellular level, NSG2 is found extensively localized to the trans-Golgi network as well as in dendritic endosomes, and was recently found to be expressed transiently at the cell surface (Yap et al., 2017). Interestingly, NSG2 also contains a Secretogranin III domain as well as an atypical EGF-like motif (Sabéran-Djoneidi et al., 1995), making it a good candidate for regulating signaling and exocytosis. However, while much is known about the spatial distribution of NSG2 in dendritic endosomes, its role in neuronal function has remained elusive.

One major function attributed to both NSG1 and NSG3 is the regulated trafficking of the AMPA-type glutamate receptors (AMPARs) at postsynaptic densities (PSDs), (for review, see Yap and Winckler, 2012; Muthusamy et al., 2015). For instance, NSG3 is found in RAB5^+^/EEA1^+^ early endosomes, interacts with AP2 and AP3, and is critical for clathrin-mediated endocytosis of AMPARs (Xiao et al., 2006; Muthusamy et al., 2012, 2015). Knock-out of NSG3 impairs long-term depression (LTD)-mediated AMPAR endocytosis, while overexpression reduces GluA1 and GluA2 surface expression and impairs performance on a fear extinction paradigm (Xiao et al., 2006; Kruusmägi et al., 2007; Davidson et al., 2009; Vazdarjanova et al., 2011). NSG1, on the other hand, is colocalized to a limited extent with the early endosome marker RAB5 and the recycling endosome marker RAB11 (Steiner et al., 2002; Yap et al., 2017). Overexpression of the C-terminal tail of NSG1 acts as a dominant negative and prevents recycling of GluA1/2-containing AMPARs following NMDA-mediated internalization (Alberi et al., 2005; Steiner et al., 2005). Together these proteins play complementary roles in regulating internalization (NSG3) and recycling (NSG1) to promote AMPAR sorting between intracellular compartments and the plasma membrane during synaptic plasticity at established synapses. However, the predominant expression of NSG2 during early periods of development (Sabéran-Djoneidi et al., 1995; Barford et al., 2017) led us to consider whether it may function during early periods of synaptogenesis. Further, while much is known about AMPAR trafficking at mature synapses, less is known regarding the mechanisms by which AMPARs are trafficked during synaptogenesis.

Here, we report that NSG2 is a critical regulator of neuronal AMPAR surface expression in hippocampal cultures during early periods of neuronal development. In agreement with previous reports (Yap et al., 2017), we found NSG2 strongly localized to the trans-Golgi network as well as in a punctate pattern selectively in developing MAP2^+^ dendrites, but is excluded from axons. In addition, a significant proportion of NSG2 punctae colocalized with synaptic markers including AMPAR subunits. Co-immunoprecipitation (Co-IP) and capillary immunoelectrophoresis experiments demonstrated a physical interaction with both GluA1 and GluA2. Overexpression and CRISPR-mediated knock-out of NSG2 altered AMPAR surface expression as well as AMPAR-mediated postsynaptic currents (PSCs). These data are the first to demonstrate a role for NSG2 in the regulation of AMPAR surface expression and implicate NSG2 as a potential partner of AMPARs in regulated endosomal trafficking in developing dendrites.

## Materials and Methods

### Cell culture, transfection, and transduction

HEK293T cells and Neuro-2a cells were maintained in DMEM supplemented with 10% fetal bovine serum (FBS) and 1× penicillin/streptomycin (Pen/Strep; Thermo Fisher Scientific). Cells at 90% confluence were passaged every 3 d using 0.05% trypsin (Thermo Fisher Scientific). HEK293T cells were transfected using the calcium phosphate method with 10 µg plasmid DNA. Neuro-2a cells were transfected using Lipfectamine 3000 (Thermo Fisher Scientific) as per manufacturer’s recommendation. Hippocampal pyramidal neurons were derived from postnatal; P0–P1 C57Bl/6J pups (The Jackson Laboratory) of either sex. Briefly, brains were isolated, and the hippocampus was dissected in ice-cold Hanks’ balanced salt solution (HBSS) solution (Sigma) supplemented with 20% FBS and NaHCO_3_ (4.2 mM), HEPES (1 mM; Sigma); pH 7.4. Dissected hippocampi were digested for 10 min with 0.25% trypsin (Thermo Fisher Scientific). Tissue was washed and dissociated using fire polished Pasteur pipettes of decreasing diameter in ice cold HBSS containing DNase (1500 U; Sigma). The cells were pelleted, resuspended in plating media and plated at a density of 4–5 × 10^5^ cells/12-mm coverslip (Electron Microscopy Sciences) coated with poly-Ornithine (0.1 mg/ml; Sigma, catalog #4638) and laminin (5 μg/ml; Thermo Fisher Scientific). Cells were allowed to adhere for 20 min before addition of 0.5 ml of plating media containing Neurobasal supplemented with 1× B27, 2 mM Glutamax, 0.5 mg/ml Pen/Strep and 5% FBS (all from Thermo Fisher Scientific) for the first 24 h. Serum was eliminated from this media after 24 h and again replaced after 48 h supplemented with 4 µM cytosine 1-β-D-arabinofuranoside (Ara-C; Sigma). Neurons were fed by replacing half the volume of spent media with fresh media without serum or Ara-C every week. All animal procedures were performed in accordance with University of New Mexico-Health Science Center animal care committee’s regulations. Neurons on coverslips were transduced with lentivirus Multiplicity Of Infection (MOI 2–3) on day 4 in culture for CRISPR studies or day 7 in culture for NSG2 overexpression studies and assayed at the days indicated in results. Human pluripotent stem cells (hPSC; line WA09) were maintained in mTesR (STEMCELL Technologies) and passaged using 1 U/ml dispase (STEMCELL Technologies) as per manufacturer’s recommendation once the cells reached 80% confluence. hPSC-derived neurons (hPSNs) were differentiated from one six well plate of hPSCs. Briefly, the cells were lifted using dispase and allowed to grow in suspension in a T75 flask for 4 d in mTeSR. From day 5 to day 20, cells were grown in neural induction media (DMEM/F12; Sigma) supplemented with 1× concentrations of N2, Glutamax, and Pen/Strep (Thermo Fisher Scientific) and 2 μg/ml heparin (Sigma) to derive neurospheres with 2/3rd volume of media replaced every other day. On day 21, 8–10 neurospheres were plated down/12-mm glass coverslip in a 24-well dish-coated with poly-ornithine and laminin as described above and allowed to attach for a minimum of 4 h before feeding with 0.5 ml neural differentiation media [50:50 mixture of Neurobasal:DMEM/F12 supplemented with 1× concentrations of N2, Glutamax, Pen/Strep, and 10 ng/ml of cAMP (Sigma), BDNF and GDNF (Peprotech), and 100 mM ascorbic acid (Sigma)]. Half volume of media was replaced every other day until the assayed days mentioned in the Results section.

### Plasmids and lentivirus production

Episomal vectors encoding mCherry or NSG2-mCherry (NSG2-mC) were generated in house by cloning either mCherry or the open reading frame of human NSG2 (NM_015980.4) in frame with mCherry into the pCS2+ mammalian expression plasmid (kind gift from Bill Bement, University of Wisconsin). The NSG1-GFP plasmid was a kind gift from Bettina Winckler (University of Virginia). The pSIN REP5-GFP-GluA2(R) was purchased from Addgene (Addgene, catalog #24005) and the YFP-GluA1 was previously described (Sharma et al., 2006). Lentiviral constructs were generated by cloning either mCherry or NSG2-mC downstream of the Synapsin-1 promoter in the pFCK plasmid (Dittgen et al., 2004). CRISPR/Cas9 guide RNAs (gRNAs) targeting mouse NSG2 (NM_008741.4) were generated using the CRSPR design webtool (https://zlab.bio/guide-design-resources). gRNAs (MsNSG2#1: 5’- GCGTGATGAGAGGGACGGTC-3′ and MsNSG2#2: 5′-CGTCCCTCTCATCACGCCCT-3′) were cloned into pL-Crispr.EFS.GFP (Addgene, catalog #57818) into the BsmBI site as previously suggested (Heckl et al., 2014). Lentivirus were produced in HEK293T cells using calcium phosphate transfection of a 15 μg total mixture of lentiviral DNA and packaging plasmids psPax2 and pMD2.G (gifts from Didier Trono, Addgene, catalog #12260 and catalog #12259, respectively) at a ratio of 3:2:1 for lentivirus production; 36–48 h post-transfection, lentivirus-containing media were harvested and concentrated using Lenti-X concentrator (Clontech) as per manufacturer’s recommendations. Pellets were resuspended in ice cold DMEM, aliquoted, and frozen at −80°C till use.

### Cell and whole-brain lysates

HEK293T cells were lysed 48 h post-transfection by incubating cells for 30 min in ice-cold lysis buffer containing: 50 mM Tris base, 150 mM NaCl, 1 mM EDTA, 1% Triton X-100, and 1× protease inhibitor cocktail (Thermo Fisher Scientific); pH 7.4. Whole-brain homogenate was prepared by harvesting mouse brains from P0 pups and subjecting them to a brief ice-cold PBS wash. Brains were then homogenized by sonication (5 × 10 s) in ice cold lysis buffer using a sonic dismembrator (Fisher Scientific, model F60) with output power set at 1. Protein amounts in the supernatant was quantified using the BCA assay (Thermo Fisher Scientific) and was aliquoted and frozen at –80°C until use after cold centrifugation at 10,000 × *g* for 15 min.

### Co-IP

For AMPAR Co-IP 2 mg total protein was incubated with 4 μg of either mouse anti-GluA1 (Clone RH95; Millipore), mouse anti-GluA2 (Clone 6C4; Millipore), or control mouse IgG1 (Clone G3A1; Cell Signaling Technology) antibodies overnight at 4°C, with gentle rocking. Immune complexes were precipitated for 2 h at 4°C using 20 μl protein A/G agarose beads (Santa Cruz Biotechnology). For NSG2-mC Co-IP, 1.5 mg total protein was incubated with either 50 μl anti-RFP mAb-agarose (MBL International Corporation) or RFP-Trap_M (ChromoTek Inc.) according to manufacturer’s instructions. Agarose beads from the above reactions were washed and denatured in 50 μl 1% SDS, and 20 μl was loaded on an SDS-PAGE gel for Western blotting or 2 μl of the supernatant was subject to capillary electrophoresis.

### Western blotting and capillary electrophoresis

SDS denatured proteins were processed via SDS-PAGE and transferred to FL-PVDF membranes (Li-COR) for traditional immunoblotting. For Western blotting primary antibodies used were rabbit anti-NSG2 (1:500; Abcam), mouse anti-β-actin (1:5000; Clone AC-15; Thermo Fisher Scientific), and mouse anti-GAPDH (1:500; Clone 6C5; Thermo Fisher Scientific). Secondary antibodies used for detection of primary antibodies were goat anti-mouse 800CW (1:15,000; Li-COR) and goat anti-rabbit 680RD (1:15,000; Li-COR), and blots were scanned using the Odyssey infrared imager and acquired on the Image Studio Lite software suite (version 3.1, Li-COR). Capillary electrophoresis was performed on the fully automated Wes system (ProteinSimple) following the manufacturer’s recommendations. Briefly, 0.5 μg protein lysate (Input lanes) or 2 μl protein (IP lanes) were mixed with 2 μl of the 5× Master mix containing SDS and DTT. The samples and protein standard were boiled at 95°C for 5 min. The samples were dispensed into microplates containing blocking buffer, primary and secondary antibody and wash buffer in independent wells for sequential processing. The plate was briefly spun and loaded into the instrument for electrophoretic separation of proteins in capillary tubes containing a 12- to 250-kDa separation matrix. The chemiluminescence based electrophoretogram was autogenerated and digitally-rendered bands were generated from the chemiluminescent peaks using the Compass software (ProteinSimple). For the Wes, primary antibodies were rabbit anti-NSG2 (1:300), mouse anti-GluA1 (1:100), and mouse anti-GluA2 (1:50). HRP-conjugated secondary anti-mouse and anti-rabbit antibodies were used at the predefined concentrations provided by the manufacturer.

### T7 endonuclease I assay

Neuro-2a cells were transfected with either the control or CRISPR NSG2 KO gRNA plasmids and allowed to express the constructs for 48–60 h. The cells were then harvested and a purified GFP^+^ cells population was obtained by flow cytometry. The genomic DNA from the GFP^+^ cell population was extracted using a commercially available kit (Zymo Research); 100 μg of genomic DNA was used as a template for PCR amplification of a fragment surrounding the putative gRNA cleavage site using the following primer pair: Fwd 5’-TCCCCGGACAATGGGAATCATG-3’ and Rev 5’-GTGGCTGGAAGAATGAAAGGAT-3’. Amplicons were then subjected to a single cycle of denaturation and renaturation to generate heteroduplex molecules containing mismatches which could be recognized and cleaved using the T7 Endonuclease I enzyme (New England Biolabs). The products of the reaction were resolved on a 2% agarose gel containing 1× gel red stain (Biotium) and imaged on a gel documentation system (Bio-Rad). The relative band intensities of the cut fragments to the uncut fragment were used to calculate the gRNA-mediated cleavage efficiency.

### Immunocytochemistry

Neurons on coverslips were fixed with 4% paraformaldehyde/4% sucrose for 15 min, rinsed three times for 5 min in PBS (Sigma), and permeabilized using 0.2% Triton X-100 for 10 min (except when staining for surface GluA1 and GluA2). Cells were blocked with 10% donkey serum in PBS for 1 h, followed by an overnight incubation of primary antibody in 5% donkey serum at 4°C. Primary antibodies consisted of rabbit anti-NSG2 (1:500; Abcam), goat anti-NSG1 (1:400; Everest Biotech), mouse anti-GFAP (1:1000; Neuromab), chicken anti-β_III_-tubulin (1:500; Millipore), rabbit anti-Homer1 (1:1000; Synaptic Systems, GmbH), guinea pig anti-Homer1 (1:200; Synaptic Systems), mouse anti-Synapsin-1 (1:2000; Synaptic Systems), chicken anti-MAP2 (1:5000; Biolegend), mouse anti-SMI312 (1:1000; Biolegend), mouse anti-PSD95 (Clone 7E3, 1:100; Thermo Fisher Scientific), and antibodies targeting the N terminus of GluA1 and GluA2 (1:100; see above, Co-IP). Following primary antibody incubation, cells were washed thrice with PBS and incubated for 1 h with secondary antibody in 5% donkey serum. Conjugated secondary antibodies used were: DyLight 488, 550, and 647 (1:1000; Thermo Fisher Scientific), donkey anti-guinea pig CF555 and goat anti-chicken CF647 (both at 1:500; Sigma). Cells were washed with PBS and then in some cases treated with DAPI (1:10,000 in PBS; Thermo Fisher Scientific), followed by three washes with 1× PBS, and mounted on superfrost slides on Fluoromount-G as an anti-quenching reagent (Southern Biotech). In our hands, the GluA1 antibody described above did not yield much success with hPSNs. Therefore, we conducted live staining for surface GluA1 on hPSNs with a custom developed rabbit anti-GluA1 targeting the extracellular epitope of GluA1 (1:200; a kind gift from Dr. Matthew Kennedy, University of Colorado Denver). The cells were incubated live with the antibody for 15 min before three brief PBS washes and fixation with 4% PFA. The postfixation treatments and secondary antibody incubations were same as described above.

### Confocal imaging and analysis

Confocal z-stacks were acquired on the Zeiss LSM800 airyscan confocal microscope using the 63x/1.40NA Oil objective. Sequential frame acquisition was set to acquire an average of 10 planes per stack at 16 bit and a minimum of 1024 × 1024 resolution. Channel gain settings were optimally adjusted to minimize saturation of punctae and were maintained across experimental groups. Unmodified images were used for all analyses and linear scaling was applied on images only for presentation purposes using Zen Black 2.3 (https://www.zeiss.com/microscopy/us/downloads/zen.html). Fluorescent signal colocalization on the single planes from a stack and quantification of punctae number was performed using the colocalization plugin for ImageJ ComDet version 0.3.4 (https://github.com/ekatrukha/ComDet) as previously described (Esteves da Silva et al., 2015). Punctae integrated fluorescence intensity over area measurements were performed with ImageJ 1.48v (https://imagej.nih.gov/ij/). For each experimental group an average of >200 μm dendrite was quantified for 8–10 images per experiment and repeated for a total of three biological replicates.

### Electrophysiology

Whole-cell patch-clamp recordings were performed a previously described (Weick et al., 2013) with minor modifications. The extracellular solution was a modified HBSS that contained: 140 mM NaCl, 3 mM KCl, 2 mM CaCl_2_, 1 mM MgCl_2_, 15 mM HEPES, and 23 mM glucose; pH 7.4, 300 mOsm. Recording pipettes with resistances of 3–5 MΩ were filled with an intracellular recording solution containing the following: 121 mM K-gluconate, 20 mM KCl, 2 mM MgCl_2_, 10 mM EGTA, 10 mM HEPES acid, 2 mM Mg^2+^-ATP, and 0.2 mM Na^+^-GTP; pH 7.2, 290 mOsm. Pharmacological antagonists picrotoxin (50 μM; Tocris), TTX (1μM; Tocris), and AP5 (25 μM; Sigma) were bath applied in the external solution. Each experiment consisted of 10–13 cells per group, and experiments were repeated three times on different cultures at the same time points indicated in the results. Neurons were visualized using an Olympus Optical BX51WI microscope (Olympus Corp.) with differential interference contrast optics at 40×. Recordings were obtained using a MultiClamp 700B amplifier (Molecular Devices), filtered at 4 kHz and sampled at 100 kHz using a Digidata 1322A analog-to-digital converter (Molecular Devices). Whole-cell capacitance was fully compensated but series resistance was not compensated. Access resistance was monitored before and after recordings, and cells with resistances >20 MΩ at either point were discarded from analyses. Miniature EPSCs (mEPSCs) were measured using a holding potential of –70 mV, while outward potassium currents were elicited by a voltage-step protocol from –50 to +50 mV, and all recordings were performed at 32°C. Step protocols were used to verify the lack of inward sodium current in all cells used for subsequent analysis. Data were stored on a computer hard disk and PSCs were analyzed using MiniAnalysis software (Synaptosoft), while potassium currents were analyzed using Clampfit v. 10.0 (Molecular devices). The cells used for mEPSC recordings for NSG2 knock-out (control GFP and CRISPR NSG2 KO) were injected with Lucifer yellow (10 mM; Thermo Fisher Scientific) included in the internal solution. After recordings cells were immediately fixed and stained for NSG2 as described above (see above, Immunocytochemistry). Only cells showing a lack of NSG2 (indicating NSG2 KO) were included for subsequent analysis.

### Statistical analyses

Student’s *t* tests were used to determine whether mean differences between groups (e.g., control mCherry vs NSG2-mC) were significant and were considered significant a priori if *p* < 0.05. The number of samples used for statistical analyses (*n*) refers to the number of cells assayed per group accumulated from three independent biological replicates for all imaging-based assays and electrophysiology experiments. For analysis of synaptic and NSG2 punctae, *n*’s are still reported by the criteria above but at least 1500 total punctae were included across cells/replicates. Data are reported as mean ± SEM.

### Data availability

Data generated and analyzed in this study are available from the corresponding author on request.

## Results

### NSG2 is localized to PSDs of excitatory synapses

**Figure 1.**
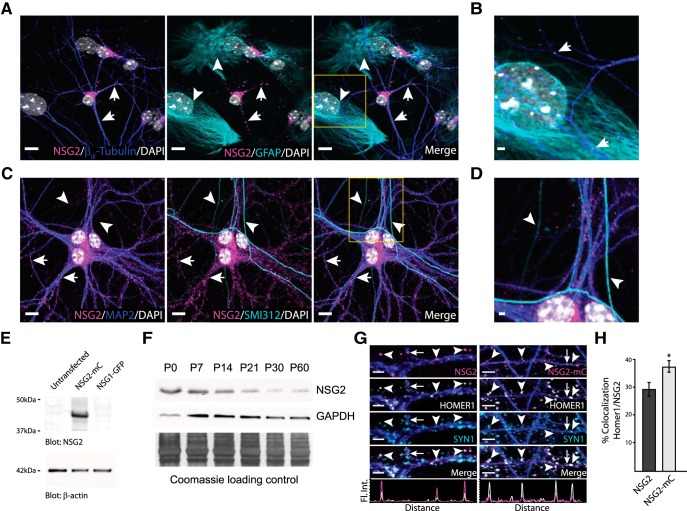
A subset of NSG2 punctae localize to PSDs of excitatory synapses. ***A***, Representative confocal images of primary hippocampal neurons (DIV4) illustrate a perinuclear as well as punctate NSG2 expression pattern (magenta; arrows) in β_III_-tubulin**^+^** neurons (blue). No NSG2 expression was observed in GFAP**^+^** astrocytes (cyan; arrowheads). The punctate expression pattern of NSG2 was found in all neurons tested up to DIV30 (Extended Data Fig. 1-1). ***B***, Magnified image of yellow box in the merge panel of ***A***. NSG2 puncta (magenta; arrows) are present only within β_III_-tubulin**^+^** neurons (blue). ***C***, Immunofluorescent images illustrate NSG2 (magenta; arrows) punctae exclusively in MAP2**^+^** (blue) dendrites and absence of NSG2 punctae in SMI312**^+^** axons (cyan; arrowheads) at DIV14. ***D***, Magnified image of yellow box in the merge panel of ***C***. ***E***, Representative Western blotting image demonstrating the specificity of the NSG2 antibody in detecting NSG2 (NSG2-mC; top panel, ∼48-kDa band; middle lane) but not the closely-related protein NSG1 (NSG1-GFP; top panel, ∼48 kDa). Bottom panel shows the β-actin loading control. ***F***, Representative Western blotting image demonstrating the expression profile of NSG2 from mouse whole-brain lysate during development (top panel, ∼19-kDa band). While GAPDH was used as a loading control (middle panel), it shows variability across development. The blot was also stained with Coomassie (bottom panel) to demonstrate equal protein loading. ***G***, Confocal images showing endogenous NSG2 (left panels) and overexpressed NSG2-mC (right panels) colocalized with HOMER1^+^ punctae (arrowheads; HOMER1 panel and merge). Endogenous NSG2 and NSG2-mC were frequently found adjacent to Synapsin1^+^ punctae (e.g., leftmost arrowheads). Individual panels for HOMER1^+^ (white) and SYN1^+^ (cyan) are shown for clarity. The colocalization profile of NSG2 or NSG2-mC (magenta trace) with HOMER1 (white trace) for the indicated punctae are indicated in the bottom panels. Some NSG2 punctae were not found colocalized to either HOMER1 or SYN1 punctae (arrow). ***H***, Quantification of colocalization of Homer1 with either endogenous NSG2 or NSG2-mC. Bars represent mean ± SEM for *n* = 10 for each group; *p = 0.024. Scale bar = 10 μm (***A***, ***B***), 2 μm (***A***, ***D***, ***G***; left panels), or 4 μm (***G***; right panels).

NSG2 is one of the most highly upregulated and abundant mRNAs during early neuronal development across multiple species, including human (Kang et al., 2011; Miller et al., 2014; Barford et al., 2017; Murillo et al., 2017; Floruta et al., 2017). To determine whether NSG2 protein was similarly upregulated, we first used differentiation of cortical neurons derived from human pluripotent stem cells (hPSCs) and assayed for NSG2 using an antibody previously shown to detect the rodent protein (Barford et al., 2017). Robust NSG2 expression was observed in 98% of the β_III_-tubulin**^+^** neurons analyzed (*n* = 151/154 neurons from five independent experiments) at days 30 and 50 in culture (Extended Data Fig. 1-1*A*,*B*). These timepoints correspond to periods before and following the formation of functional synapses in hPSC-derived neurons (hPSNs), respectively. NSG2 was not found in neuroepithelial cells at day 30 that were PAX6**^+^**/MAP2**^-^** (Extended Data Fig. 1-1*C*), suggesting its upregulation following the final mitotic division. Similarly, NSG2 was detected by 4 d *in vitro* (DIV4) in cultured mouse hippocampal neurons (Fig. 1*A*), an early time point before the creation of functional synapses (Grabrucker et al., 2009). NSG2 remained robustly expressed in all hippocampal neurons examined (*n* = 391/391; from six independent cultures) at all time points assayed up to DIV30 (Extended Data Fig. 1-2). Similar to previous reports, NSG2 was found robustly aggregated in perinuclear regions consistent with localization to the Golgi apparatus (Sabéran-Djoneidi et al., 1995) as well as in distinct punctae throughout β_III_-tubulin**^+^** neurites (Fig. 1*A*,*B*, yellow box magnified; Extended Data Fig. 1-1, blue, arrows). At DIV4, NSG2 was absent from GFAP**^+^** astrocytes in regions that did not have overlapping β_III_-tubulin**^+^** neurites (Fig. 1*A*,*B*, yellow box magnified, cyan, arrowheads). This was also evident in cultures of human neurons and glia at day 50 (Extended Data Fig. 1-3*A*), supporting the neuron-specific nature of NSG2 across species. At DIV14 in mouse neurons, the number of NSG2 punctae were dramatically increased as neuronal arbors became more elaborate (Fig. 1*C*). At this time point, NSG2 punctae were restricted to the somatodendritic compartment as MAP2^+^ dendrites showed robust staining (Fig. 1*C*,*D*, yellow box magnified; Extended Data Fig. 1-3*B*, blue, arrows), whereas SMI-312**^+^** axons were devoid of NSG2 (Fig. 1*C*,*D*, yellow box magnified; Extended Data Fig. 1-3*B*, cyan, arrowheads). Together, these data support the neuron-specific nature of NSG2 and show its punctate localization restricted to somatodendritic arbors of human and mouse neurons.

10.1523/ENEURO.0292-18.2018.f1-1 Extended Data Figure 1-1 NSG2 is robustly expressed in hPSNs. ***A***, Representative confocal immunofluorescence images of day 30 hPSNs showing the expression and distribution of NSG2 (magenta; arrows) in β_III_-tubulin**^+^** neurons (cyan). DAPI**^+^** nuclei are indicated in white. ***B***, Representative confocal images of day 50 hPSNs showing the expression and distribution of NSG2 (magenta; arrows) in β_III_-tubulin**^+^** neurons (cyan). DAPI**^+^** nuclei are indicated in white. ***C***, Representative confocal images of hPSNs illustrate NSG2 (magenta; arrows) expression restricted to MAP2**^+^** (blue) neurons and absent from Pax6**^+^** neural progenitors in day 30 hPSN cultures. Nuclear Pax6**^+^** (cyan; arrowheads) colocalized with DAPI**^+^** nuclei (white; arrowheads). Scale bars = 10 μm. Download Figure 1-1, EPS file.

**Figure 2.**
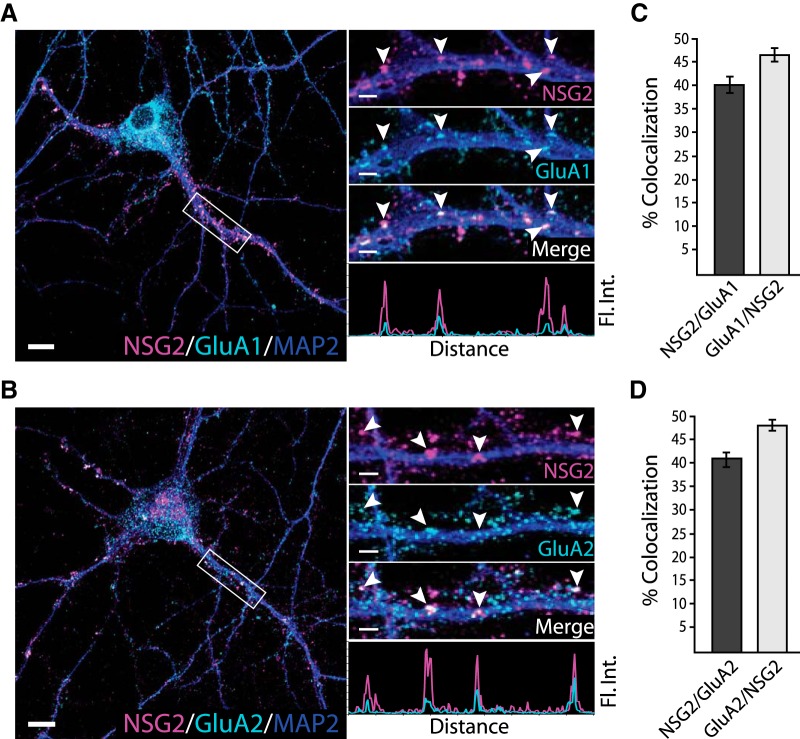
NSG2 colocalizes with AMPAR subunits GluA1 and GluA2. ***A***, ***B***, Representative confocal images of primary hippocampal neurons at DIV15 showing perinuclear and punctate NSG2 (magenta) in MAP2^+^ (blue) neurites along with surface expressed GluA1 punctae (***A***, cyan), and GluA2 punctae (***B***, cyan). Separated color panels for individual markers from the boxed regions have been magnified for clarity (right panels). Lower right panels in ***A***, ***B*** show the colocalization profile for NSG2 (magenta)/GluA1 (cyan, panel ***A***) and NSG2 (magenta)/GluA2 (cyan, panel ***B***) across the indicated punctae (arrowheads). ***C***, ***D***, Quantification of colocalization for NSG2 with surface GluA1 punctae (6423 NSG2 and 5392 GluA1 puncta from 10 neurons) and NSG2 with surface GluA2 punctae (6874 NSG2 and 5963 GluA2 puncta from nine neurons), respectively, from three independent cultures. Scale bars = 10 μm (***A***, ***B***; left panels) and 2 μm (***A***, ***B***; right panels).

10.1523/ENEURO.0292-18.2018.f1-2 Extended Data Figure 1-2 NSG2 remains expressed in mouse hippocampal neurons at DIV30. Representative confocal immunofluorescence images of primary hippocampal neurons (DIV30) showing the expression and distribution of NSG2 (magenta; arrow) in β_III_-tubulin**^+^** neurons (cyan). DAPI**^+^** nuclei are indicated in white. Scale bars = 5 μm. Download Figure 1-2, EPS file.

**Figure 3.**
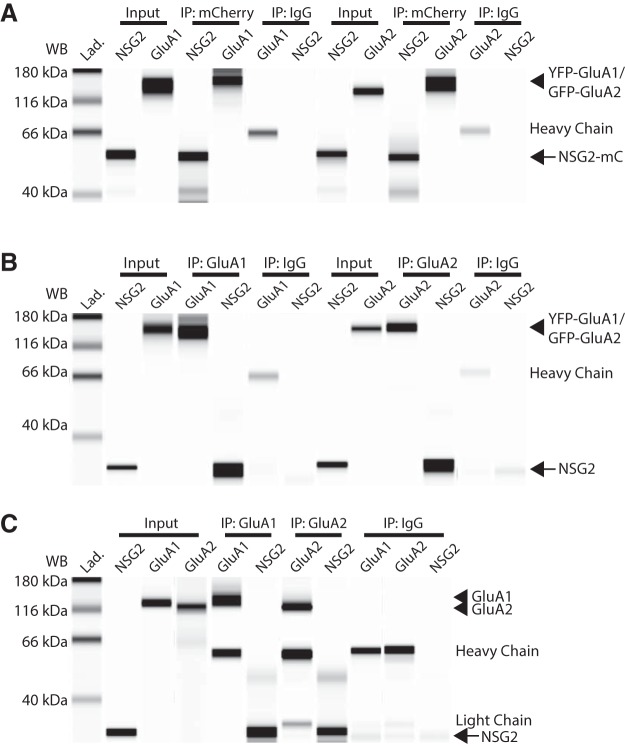
NSG2 interacts with AMPAR subunits GluA1 and GluA2. ***A***, Co-IP of overexpressed, fluorophore-conjugated proteins. Representative digitized Western blottings from the automated Western blotting system (see Materials and Methods) showing that using the anti-RFP antibody to pull down NSG2-mC (IP: RFP, arrow) could Co-IP both YFP-GluA1 and GFP-GluA2 (IP: mCherry, arrowhead). A control IgG antibody did not IP nor detect any target proteins (IP: IgG); the heavy chain of mouse and anti-RFP was detected by the anti-mouse secondary antibody used to detect mouse anti-GluA1/2. NSG2-mC (arrow), YFP-GluA1, and GFP-GluA2 (arrowhead) were detected in the input (input). Traditional Western blotting techniques were performed to confirm that GluA2 co-immunoprecipitates with NSG2-mC (Extended Data Fig. 3-1). Samples were taken from HEK293T cells co-expressing NSG2-mC and either YFP-GluA1 or GFP-GluA2. ***B***, Immunoprecipitation of overexpressed, unlabeled NSG2 with fluorophore-conjugated proteins with antibodies directed toward endogenous protein epitopes. Anti-GluA1 and anti-GluA2 antibodies immunoprecipitated YFP-GluA1 and GFP-GluA2, respectively (IP: GluA1 and IP: GluA2, arrowhead). Unlabeled NSG2 (arrow) coexpressed in HEK293T cells was also detected using these same conditions, confirming the Co-IP; control IgG did not IP nor detect any target proteins (IP: IgG). NSG2, YFP-GluA1, and GFP-GluA2 are detected in the input (input; GluA1; GluA2, arrowhead; NSG2, arrow). Lower bands in NSG2 lanes of control IgG pull down conditions is thought to be non-specific is found in all similar reaction conditions and migrates faster than NSG2. ***C***, Immunoprecipitation from *in vivo* samples (whole-brain lysate of P0 mouse). Anti-GluA1 and anti-GluA2 antibodies immunoprecipitated endogenous GluA1 and GluA2 (IP: GluA1 and IP: GluA2, arrowhead) as well as endogenous NSG2 (arrow). All target proteins were detected in the input lanes (input; GluA1 and GluA2, arrowhead; NSG2, arrow). Control mouse IgG antibody did not pull down GluA1, GluA2, or NSG2 (IP: IgG). As expected, heavy chain and light chains from antibodies used for pull down were detected in most Co-IP conditions (all IP lanes).

10.1523/ENEURO.0292-18.2018.f1-3 Extended Data Figure 1-3 NSG2 expression in hPSNs is neuron-specific and somatodendritic. ***A***, Representative confocal images of hPSNs illustrate NSG2 expression is perinuclear (magenta; arrows) and localized to punctae distributed in MAP2**^+^** (blue) dendrites; NSG2 punctae are absent in GFAP**^+^** astrocytic processes (cyan; arrowheads) at day 50. Far right panel shows magnified image of yellow box in merge. ***B***, Representative confocal images of hPSNs illustrate NSG2 punctae (magenta; arrows) exclusively in MAP2**^+^** (blue) dendrites but absent from SMI312**^+^** axons (cyan; arrowheads) at day 50. Far right panel shows magnified image of yellow box in merge. DAPI**^+^** nuclei are indicated in white. Scale bars = 10 μm. Download Figure 1-3, EPS file.

In contrast to previous reports suggesting that NSG2 protein is nearly absent in brain following early postnatal development (Sabéran-Djoneidi et al., 1995), Barford et al., recently showed that expression of NSG2 was maintained in cortex up to the period of adolescence (P16) but was absent from cerebellum in adults (P60; Barford et al., 2017). Due to sequence homology between NSG family members we determined whether the antibody used for immunostaining was specific for NSG2. Western blot analysis was used to validate the specificity of the NSG2 antibody, which revealed a band of ∼47 kDa in lysates taken from HEK293T cells overexpressing NSG2 (19 kDa) linked to the mCherry (28 kDa) fluorophore (NSG2-mC; Fig. 1*E*, upper panel, middle lane). However, no bands were detected in lysates taken from HEK293T cells overexpressing NSG1 (21 kDa) linked to GFP (27 kDa; Fig. 1*E*, upper panel, right lane) or untransfected HEK293T cell lysates (Fig. 1*E*, upper panel, left lane). β-Actin served as a loading control (Fig. 1*E*, lower panel). Further, to determine the expression profile of endogenous NSG2 protein over time, we performed Western blotting of mouse whole-brain lysates. We found that while NSG2 has its greatest expression during the perinatal period and is gradually downregulated thereafter, it continues to be expressed even in adult animals (P60; Fig. 1*F*) consistent with recent studies (Barford et al., 2017).

Because of the punctate nature of NSG2 expression in dendrites, we determined whether NSG2 localized to synapses. In DIV14–DIV16 hippocampal neurons a portion of endogenous NSG2 (endo-NSG2) punctae was independent of both presynaptic Synapsin-1 (SYN1) and postsynaptic HOMER1 (Fig. 1*G*, arrow), whereas strong synaptic localization was observed for a subset of endo-NSG2 punctae (Fig. 1*G*, left panels, arrowheads). Quantification of colocalization showed that ∼30% of HOMER1 punctae colocalized with NSG2. By comparison, NSG2 punctae were frequently found adjacent to, but not colocalized with SYN1 punctae (Fig. 1*G*, left arrowhead). In fact, some endo-NSG2 punctae were found colocalized with HOMER1 that were not adjacent to SYN1 punctae (Fig. 1*G*, left panels, far right arrowhead). Similar results were found with overexpressed NSG2-mC (Fig. 1*G*, right panels), indicating this construct behaves similarly to the endo-NSG2 protein. Interestingly, the percentage of HOMER1 punctae that colocalized with NSG2-mC was significantly increased compared to that for endo-NSG2 (Fig. 1*H*; endo-NSG2: 29.2 ± 2.5%; NSG2-mC: 37.0 ± 2.0%; *p = 0.024). This happened despite the fact that the total densities of HOMER1 punctae for control and NSG2-mC were not significantly different (mCherry: 126 ± 7 punctae/100 µm; NSG2-mC: 133 ± 10 punctae/100 µm; *p* = 0.6). Thus, the increased colocalization between NSG2-mC and HOMER1 is likely a result of NSG2-mC entering HOMER1**^+^** synapses that are not occupied by endo-NSG2. Taken together, these data suggest that NSG2 is present in a subset of excitatory synapses and is likely restricted to the PSD rather than presynaptic terminals. Furthermore, overexpressed NSG2-mC was not sufficient to drive formation of new HOMER1^+^ PSDs but was able to enter new sites where endo-NSG2 was likely absent.

### NSG2 interacts with AMPAR subunits GluA1 and GluA2

To determine whether NSG2 colocalizes with functional AMPAR-containing synapses, we used surface labeling with antibodies directed toward the extracellular N-termini of the GluA1 and GluA2 subunits. Figure 2*A*,*B* shows representative confocal images of neurons (left panels) as well as individual neurites (right panels) from boxed regions. Results for both GluA1 and GluA2 revealed robust colocalization with individual NSG2 punctae (Fig. 2*A*,*B*, right panel arrowheads). Pooled data revealed that ∼40% of NSG2 punctae colocalized with either the GluA1 (Fig. 2*A*,*C*; NSG2/GluA1: 40.1 ± 1.6%) or GluA2 subunits (Fig. 2*B*,*D*; NSG2/GluA2: 41.0 ± 1.5%). Reciprocally, over 45% of GluA1 and GluA2 surface labeled punctae colocalized with NSG2, suggesting that nearly half of all AMPAR-containing PSDs contained NSG2 protein at DIV15 (Fig. 2*C*,*D*; GluA1/NSG2: 46.2 ± 1.2%; GluA2/NSG2: 48.0 ± 0.9%). It is noteworthy that the percentage of colocalization between AMPAR subunits and NSG2 was slightly greater than that of HOMER1/NSG2, indicating that NSG2 is somewhat selective for AMPAR-containing PSDs. We additionally verified that overexpressed NSG2-mC colocalized with human GluA1 subunits in hPSNs at day 50. While these data revealed significant colocalization of NSG2 with surface GluA1 in hPSNs (Extended Data Fig. 2-1), the inconsistent maturational state of individual hPSNs (Johnson et al., 2007) makes characterization of NSG2 function more difficult. Thus, all future experiments were conducted on cultured mouse hippocampal neurons.

10.1523/ENEURO.0292-18.2018.f2-1 Extended Data Figure 2-1 NSG2 colocalizes with AMPAR subunits GluA1 in hPSNs. Representative confocal images of hPSNs at day 50 overexpressing NSG2-mC showing perinuclear and punctate NSG2 (magenta) in MAP2^+^ (blue) dendrites along with surface expressed GluA1 punctae (cyan). Separate magnified panels for each marker indicated by the boxed region are provided for clarity (right panels). Colocalized NSG2 punctae (magenta) and surface GluA1 (cyan) are indicated by arrows while non-colocalized proteins are indicated by arrowheads. Scale bars = 10 μm (left panel) and 2 μm (right panels). Download Figure 2-1, EPS file.

We next determined whether NSG2 physically associates with AMPAR subunits GluA1 and GluA2. We first performed Co-IP from HEK293T cells co-expressing the fusion proteins NSG2-mC (∼45 kDa) and either YFP-GluA1 or GFP-GluA2 (∼128 and 125 kDa, respectively). Using an anti-mCherry monoclonal antibody we immunoprecipitated NSG2-mC and probed for YFP-GluA1 and GFP-GluA2 using antibodies specific for each subunit. Figure 3*A* shows data from a capillary immunoelectrophoresis that demonstrate NSG2-mC was able to Co-IP both GluA1 and GluA2 independently (lanes marked IP: mCherry) when the two proteins were co-expressed in HEK293T cells. An IgG control antibody did not IP either NSG2 or AMPAR subunits demonstrating the specificity of the reaction (Fig. 3*A*; IP: IgG); the heavy chain of the control mouse IgG antibody is detected by the anti-mouse secondary antibody used to detect GluA1 and GluA2. To further validate our findings, we used the traditional Co-IP/SDS-PAGE to probe for a NSG2-mC and GluA2 interaction (Extended Data Fig. 3-1, middle lane). In this case, 24-h incubation of lysates taken from HEK293T cells that expressed either protein separately showed significantly reduced Co-IP, suggesting that their co-expression in a cellular context may be necessary to drive NSG2-GluA2 interactions (Extended Data Fig. 3-1, right lane). We also performed the complementary experiment to verify the interaction of overexpressed proteins. Immunoprecipitation of either GluA1 or GluA2 using antibodies previously described (Schwenk et al., 2014) could Co-IP both overexpressed NSG2 (Fig. 3*B*, arrow) as well as endogenous NSG2 (Fig. 3*C*, arrow). In this case we used whole-cell lysates from HEK293T cells co-expressing either GFP-GluA2 or YFP-GluA1 with unlabeled NSG2 (∼19 kDa) to demonstrate that non-specific interactions between the fluorophores played no role in the interactions. In Figure 3*B*, the lanes marked IP: GluA1 and IP: GluA2 indicate that both GluA1 and GluA2 were able to Co-IP NSG2 (lower band, arrow). Finally, we asked whether the interaction between NSG2 and GluA1 and GluA2 subunits of AMPARs occurs *in vivo*. Utilizing whole-brain protein lysates from P0 mouse we found that immunoprecipitation of either GluA1 or GluA2 AMPAR subunits pulled down endo-NSG2 (Fig. 3*C*; lanes marked IP: GluA1 and IP: GluA2). Non-specific mouse IgG antibody did not pull down either AMPARs or NSG2 (Fig. 3*A–C*; lanes marked IP: IgG). Heavy and light chains of control IgG are present similar to heavy chain in Figure 3*A*,*B*; lowest bands in “IP: IgG” group are likely non-specific as they appear in all groups and are of lower molecular weight compared to NSG2.

10.1523/ENEURO.0292-18.2018.f3-1 Extended Data Figure 3-1 NSG2 and GluA2 physically interact. Representative Western blotting image showing Co-IP of NSG2-mC and GluA2 by anti-RFP in lysates from HEK293T cells co-expressing both proteins (Co-IP RFP Trap; NSG2-mC, red bands; GFP-GluA2, green bands). The specificity of antibodies is confirmed by the absence of bands in lysates from untransfected cells (untransfected) and presence of bands of expected size in the input lane (input-coexpression lysate). Anti-RFP was unable to Co-IP NSG2-mC GluA2 when the two proteins were overexpressed in different HEK293T cultures, and lysates were incubated together and then subject to immunoprecipitation (*in vitro* incubation). Download Figure 3-1, EPS file.

### NSG2 expression affects AMPAR currents

**Figure 4.**
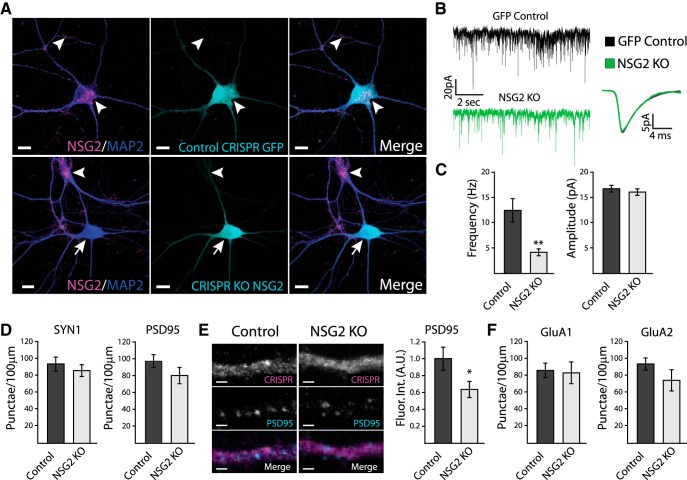
Knock-out of NSG2 decreases mEPSC frequency. ***A***, Representative confocal images of primary hippocampal neurons at DIV15 showing robust NSG2 (magenta) in MAP2**^+^** (blue) neurons transduced with control CRISPR GFP lentivirus (cyan; top panels), whereas neurons transduced with CRISPR KO NSG2 lentivirus (cyan, bottom panels) show the absence of NSG2 (arrow; bottom panels); NSG2 (magenta) is present in an adjacent neuron not transduced with the CRISPR KO NSG2 lentivirus in the same field (arrowhead; bottom panels). ***B***, Representative traces from whole-cell patch clamp recordings from neurons expressing either control CRISPR GFP (upper trace, black) or CRISPR KO NSG2 (bottom trace, green). Averaged mEPSCs from both control (black, *n* = 10) and NSG2 KO (green, *n* = 13) are shown to the right. ***C***, Pooled data revealed a significant decrease in mEPSC frequency in neurons expressing NSG2 KO compared to cells expressing control CRISPR GFP (**p = 0.001). The amplitude of mEPSCs was not significantly different between groups (*p* = 0.34). Data in the KO group were derived only from neurons devoid of NSG2 confirmed by post-recording immunostaining of Lucifer yellow injected neurons (Extended Data Fig. 4-1). NSG2 KO did not alter outward potassium I/V relationship (Extended Data Fig. 4-2). ***D***, Quantification of presynaptic marker Synapsin1**^+^** punctae (control, *n* = 10; NSG2 KO, *n* = 10; *p* = 0.46) and postsynaptic marker PSD95**^+^** punctae (control, *n* = 9; NSG2 KO, *n* = 11; *p* = 0.18). ***E***, Representative confocal images illustrate PSD95 immunofluorescence (cyan) in neurons expressing CRISPR KO NSG2 (right panels) or controls CRISPR GFP (left panels). GFP expression from both groups (top panels) is presented in grayscale for clarity. Quantification revealed a significant reduction in PSD95 fluorescence intensity in neurons expressing NSG2 KO (*n* = 11) compared to controls (*n* = 9; *p = 0.029). ***F***, Pooled data show that the number of surface GluA1^+^ punctae (left; control, *n* = 10 and NSG2 KO, *n* = 9; *p* = 0.86) and surface GluA2^+^ punctae (right; control, *n* = 10 and NSG2 KO, *n* = 9; *p* = 0.18) remained unchanged between groups. Bars represent mean ± SEM. Scale bars = 10 μm (***A***) and 1 μm (***E***).

Since NSG2 colocalizes with and is found in a complex with AMPAR subunits GluA1 and GluA2, we next asked if manipulating NSG2 protein levels would alter AMPAR surface expression as well as excitatory synaptic transmission. Two CRISPR/Cas9 gRNAs were designed using the MIT CRISPR design tool to target the mouse NSG2 locus. A T7 endonuclease cleavage assay determined that both gRNA#1 and gRNA#2 created indels in the NSG2 locus in mouse Neuro-2a neuroblastoma cells. However, the efficiency (Extended Data Fig. 4-3*C*) of gRNA#2 was significantly greater than gRNA#1, so this one was used for all experiments in primary hippocampal neurons. Transduction of control CRISPR virus and NSG2 gRNA was performed at DIV4 and neurons were analyzed at DIV16–DIV18. Figure 4*A*, upper panels, shows robust NSG2 punctae (arrowheads) in neurons transduced with control CRISPR lentivirus. In contrast, neurons transduced with the CRISPR gRNA targeting NSG2 resulted in a complete knock-out of NSG2 (Fig. 4*A*, lower panels; NSG2 KO; arrow). Note that no effect on NSG2 expression was observed in adjacent, non-transduced neurons (Fig. 4*A*, lower panels, arrowhead). Interestingly, the quantification of NSG2 KO in neurons transduced with NSG2 CRISPR virus revealed that ∼70% of neurons (*n* = 38/55, from three independent cultures) had a complete KO. To confirm specificity of CRISPR targeting, we analyzed NSG1 expression in neurons that expressed NSG2 gRNA and showed complete KO of NSG2 protein. These neurons showed no significant difference in the density of NSG1 punctae compared to neurons expressing control CRISPR constructs (Extended Data Fig. 4-3*B*; control: 346.3 ± 137.75 punctae/100 μm; NSG2 KO: 354.3 ± 134.26 punctae/100 μm; *p* = 0.97). To determine the effect of NSG2 KO on synaptic transmission we performed whole-cell patch clamp recordings on GFP^+^ neurons that received the CRISPR-NSG2 KO virus. To eliminate possible contamination from the small proportion of neurons that did not show NSG2 KO, we used Lucifer yellow injections followed by *post hoc* analysis of KO efficiency by immunocytochemistry to correlate NSG2 levels with physiologic recording data (Extended Data Fig. 4-1; only cells with undetectable NSG2 signal were used for analysis). Figure 4*B* shows representative traces of voltage-clamp recordings from neurons expressing control CRISPR (black) or NSG2 KO CRISPR virus (green). Compared to control neurons, NSG2 KO neurons showed a significant decrease in the frequency of mEPSCs, with no change in amplitude (Fig. 4*B*,*C*; frequency; control: 12.41 ± 2.31 Hz; NSG2 KO: 4.07 ± 0.68 Hz; **p = 0.001; amplitude; control: 16.71 ± 4.10 pA; NSG2 KO: 15.84 ± 4.47 pA; *p* = 0.34). These effects were specific to synaptic currents as voltage-gated potassium currents remained unchanged (Extended Data Fig. 4-2). To probe the mechanism of how NSG2 might affect mEPSCs, we performed immunocytochemical labeling of multiple synaptic proteins. NSG2 KO did not affect the density of presynaptic SYN1^+^ nor postsynaptic PSD95^+^ punctae along neurites (Fig. 4*D*; SYN1; control: 93 ± 8 punctae/100 μm; NSG2 KO: 79 ± 7 punctae/100 μm; *p* = 0.46; PSD95; control: 97 ± 7 punctae/100 μm; NSG2 KO: 80 ± 9 punctae/100 μm; *p* = 0.18). Interestingly, the fluorescence intensity of PSD95^+^ punctae was significantly decreased in NSG2 KO neurons compared to controls (Fig. 4*E*; control: 3.70 ± 0.45 A.U.; NSG2 KO: 2.57 ± 0.28 A.U.; *p = 0.04). This occurred without a corresponding change in the density of GluA1- and GluA2-containing punctae along neurites (Fig. 4*F*; surface GluA1^+^; control: 86 ± 8 punctae/100 μm; NSG2 KO: 83 ± 12 punctae/100 μm; *p* = 0.86 and surface GluA2^+^; control: 94 ± 7 punctae/100 μm; NSG2 KO: 74 ± 12 punctae/100 μm; *p* = 0.18). Together, these data indicate that the reduction in mEPSC frequency due to loss of NSG2 may be caused by a relatively subtle effect on postsynaptic scaffolding rather than a failure of AMPAR exocytosis.

10.1523/ENEURO.0292-18.2018.f4-1 Extended Data Figure 4-1 Lucifer yellow injections used to verify NSG2 KO in recorded neurons. Representative confocal images of primary hippocampal neurons (DIV15) showing the absence of NSG2 (magenta, arrow) in MAP2**^+^** neurons (blue) transduced with CRISPR KO-NSG2 lentivirus (cyan). Lucifer Yellow was injected into neurons expressing CRISPR KO-NSG2 during whole-cell patch clamp experiments (inset: Luc; yellow; Fig. 4). NSG2 (magenta) was present in MAP2**^+^** neurons not expressing CRISPR KO-NSG2 (arrowhead). Scale bars = 10 μm. Download Figure 4-1, EPS file.

10.1523/ENEURO.0292-18.2018.f4-2 Extended Data Figure 4-2 Alterations in NSG2 levels does not affect voltage-gated potassium currents. ***A***, ***B***, Current-voltage relationship plots illustrating that neither NSG2-KO (***A***, open circles) nor NSG2-mC overexpression (***B***, open circles) caused a significant change to transient potassium current elicited by voltage steps compared to controls (***A***, ***B***, black squares). ***C***, ***D***, Representative traces from plots in panels ***A***, ***B*** in response to voltage steps from –50 to +50 mV (step size, 10 mV). Download Figure 4-2, EPS file.

10.1523/ENEURO.0292-18.2018.f4-3 Extended Data Figure 4-3 Knock-out of NSG2 does not alter NSG1 levels. ***A***, Representative confocal images of primary hippocampal neurons (DIV15) showing the presence of NSG1 (white, arrowhead) in MAP2**^+^** (blue) neurons from GFP**^+^** cells transduced with either CRISPR control (cyan; top panels) or CRISPR KO-NSG2 lentivirus (cyan; bottom panels). Middle panels illustrate the presence of NSG2 (magenta, arrow) in MAP2**^+^** neurons (blue) transduced with CRISPR control (top panels) and absence of NSG2 (arrow) in MAP2**^+^** neurons (blue) transduced with CRISPR KO-NSG2 lentivirus (cyan). Scale bars = 10 μm. ***B***, Pooled data show that the density of NSG1 punctae remained unchanged between groups (bars represent mean ± SEM, *n* = 10 per group; *p* = 0.97). ***C***, Representative agarose gel showing digested fragments from mouse NSG2 DNA amplicon (arrow) were observed in cells that received either gRNA#1 or gRNA#2 + T7, but not in control or without T7. Download Figure 4-3, EPS file.

**Figure 5.**
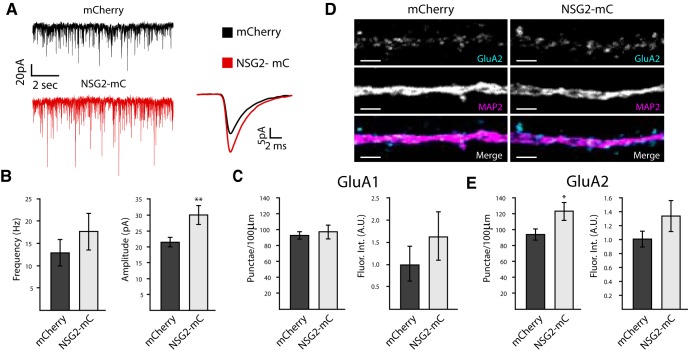
Overexpression of NSG2 increases mEPSC amplitude. ***A***, Representative traces from whole-cell patch clamp recordings from neurons expressing either mCherry alone (upper trace, black) or NSG2-mC (bottom trace, red). Averaged mEPSCs from both control (black, *n* = 11) and NSG2-mC (red, *n* = 10) are shown to the right. ***B***, Pooled data illustrate that neurons expressing NSG2-mC showed a significant increase in mEPSC amplitude compared to controls (**p = 0.01), whereas the frequency of mEPSCs was not different between groups (*p* = 0.35). ***C***, Neither GluA1^+^ punctae number (*p* = 0.58) nor relative fluorescence intensity (*p* = 0.36) were significantly different between groups (mCherry vs NSG2-mC). ***D***, Representative confocal images showing neurons expressing either mCherry alone (left panels) or NSG2-mC (right panels) stained for MAP2 (magenta) and surface GluA2 (cyan). Individual GluA2 and MAP2 expression from both groups (top two panels) are presented in grayscale for clarity. ***E***, Pooled data revealed the number of GluA2^+^ punctae was significantly increased in neurons expressing NSG2-mC (*n* = 10) compared to controls (*n* = 10; *p = 0.02), while relative fluorescence tntensity remained unchanged (*p* = 0.19). Bars represent mean ± SEM; A.U., arbitrary units. Scale bars = 2 µm.

To test whether NSG2 can augment postsynaptic targeting of AMPARs, we used overexpression of NSG2-mC, which shows nearly identical synaptic targeting as endo-NSG2 (Fig. 1*D*). Figure 5*A* shows representative traces of voltage-clamp recordings from neurons overexpressing mCherry (black) alone or NSG2-mC (red). Pooled data revealed that neurons expressing NSG2-mC showed a significant increase in mEPSC amplitude compared to those expressing mCherry alone (Fig. 5*A*,*B*; mCherry: 21.61 ± 1.43 pA; NSG2-mC: 30.22 ± 3.07 pA; **p = 0.01). In contrast to NSG2 KO, no significant difference was observed for mEPSC frequency on NSG2 overexpression (Fig. 5*B*; mCherry: 12.88 ± 2.91 Hz; NSG-mC: 17.66 ± 4.12 Hz; *p* = 0.35). Immunocytochemical analyses revealed that the intensity of GluA1 and GluA2 staining showed a trend toward an increase but that did not reach significance (Fig. 5*C*,*E*; fluorescence intensity of GluA1; mCherry: 1.00 ± 0.38 A.U.; NSG2-mC: 1.63 ± 0.54 A.U.; *p* = 0.36 and GluA2; mCherry: 1.00 ± 0.11 A.U.; NSG2-mC: 1.32 ± 0.21 A.U.; *p* = 0.19). Interestingly, while the number of surface GluA1^+^ punctae remained unchanged (Fig. 5*C*; mCherry: 92 ± 10 punctae/100 μm; NSG2-mC: 97 ± 8 punctae/100 μm; *p* = 0.58), the number of GluA2^+^ punctae was significantly increased on NSG2-mC overexpression (Fig. 5*E*; mCherry: 94 ± 6 punctae/100 μm; NSG2-mC: 123 ± 10 punctae/100 μm; *p = 0.02). These data suggest that NSG2 is sufficient to drive increased surface expression of AMPARs into existing functional synapses, and potentially to a small proportion of previously silent synapses.

## Discussion

While critical roles in endosomal trafficking and synaptic plasticity have been established for NSG1 and NSG3, no functional role for NSG2 has previously been demonstrated. Here, we present evidence that NSG2 is a novel modulator of excitatory synaptic function. We found NSG2 displays a punctate pattern of expression that is restricted from axons but found robustly throughout somatodendritic arbors. Approximately forty percentage of all PSDs that contained surface expressed AMPAR subunits also displayed colocalized NSG2 punctae. Reciprocal immunoprecipitation assays revealed that NSG2 interacts with both GluA1 and GluA2 AMPAR subunits *in vitro* and *in vivo*. CRISPR/Cas9-mediated knock-out of NSG2 in primary hippocampal neurons resulted in a significantly reduced frequency of AMPAR-mediated mEPSCs, in the absence of a reduction in total surface expressed AMPAR levels. Surprisingly, overexpression of NSG2 caused a significant increase in the amplitude of AMPAR-mediated mEPSCs that was coincident with increased surface expression of the AMPAR subunit GluA2, but did not affect frequency as in the knock-out condition. Together, these data suggest NSG2 is a novel player in excitatory postsynaptic function potentially via trafficking of AMPAR and/or associated cargo proteins (see below).

While it remains unclear as to how NSG2 modulates excitatory synaptic function specifically, the preponderance of evidence points to the regulation of AMPAR surface expression via secretory trafficking. This is supported by our current findings in conjunction with a number of findings for other NSG family members. For instance, NSG3 facilitates clathrin-mediated endocytosis of AMPARs during agonist stimulated activity that causes synaptic depression (Xiao et al., 2006; Davidson et al., 2009). Overexpression of NSG3 was shown to cause internalization of GluA1 and GluA2, decreasing surface expression levels of AMPARs. Furthermore, chronic upregulation of NSG3 in a transgenic mice was shown to impair executive cognitive function (Xiao et al., 2006; Kruusmägi et al., 2007; Davidson et al., 2009; Vazdarjanova et al., 2011), likely via alterations in AMPAR trafficking and altered LTD like processes (Myers et al., 2006; Kim et al., 2007). In contrast, NSG1 has been shown to regulate the rate of AMPAR receptor recycling within the PSD (Steiner et al., 2005; Utvik et al., 2009). While no studies report the functional consequences of full-length NSG1 overexpression, inhibition of NSG1 function via a dominant negative peptide prevented constitutive recycling of GluA1/2-containing AMPARs following NMDA-mediated internalization (Alberi et al., 2005; Steiner et al., 2005). Similarly, antisense-mediated suppression of NSG1 function severely impaired the amplitude of evoked potentials in organotypic hippocampal slices (Alberi et al., 2005). However, it is not clear whether the observed effect on amplitude was a consequence of a reduction in the number of AMPARs within individual synapses, or a reduction in the number of synapses that contained AMPARs. For example, Levy et al. (2015) demonstrate that the effect of changing levels of the MAGUK family of scaffolding proteins, specifically knock-down of PSD95 causes a reduction in AMPAR mEPSC frequency in dissociated hippocampal neurons by increasing the number of silent synapses, however without affecting mEPSC amplitudes (Levy et al., 2015). Thus, one explanation for our observations could be that in NSG2 KO neurons, a subset of synapses that would have contained NSG2 showed reduced or altered AMPAR localization that our methods were not sensitive enough to detect via immunocytochemistry. In this case, these synapses demonstrated increased failure rates or sub-threshold events which resulted in a decrease in mEPSC frequency. However, the absence of an effect on AMPAR surface expression suggests either subtle changes in AMPAR localization or an alternate mechanism. We cannot rule out a role of NSG2 on presynaptic function, although manipulation of NSG1 and NSG3 does not alter presynaptic release (Alberi et al., 2005; Davidson et al., 2009), and NSG2 is solely found in the PSD (Figs. 1, 2; Alberi et al., 2005; Steiner et al., 2005). While future studies are needed to elucidate the mechanistic details of how NSG2 is involved in regulating AMPAR trafficking, our data support a complementary role of NSG2 with that other NSG family members.

One mechanism likely involves secretion of AMPARs via post-Golgi endosomal vesicles that may contain individual or combinations of NSG family members depending on function. For instance, Yap and colleagues recently demonstrated that both NSG1 and NSG2 puncta in dendrites show approximately 60% overlapping expression with the remaining forty percentage not colocalized. Interestingly, a significant proportion of overlap was found in Rab7^+^ late endosomes traveling in a retrograde fashion which the authors conclude were likely destined for degradation. Smaller proportions of NSG1/2 puncta found in EEA1^+^ early endosomal, Rab11^+^ recycling endosomes, and Lamp1^+^ lysosomes (Yap et al., 2017). Most intriguingly, this analysis accounted for nearly all NSG1^+^ punctae, but <80% of NSG2^+^ punctae. Thus, it remains to be determined whether there is an additional pool of unique, NSG2-containing vesicles. Evidence for divergent trafficking mechanisms stems from the fact that NSG1 was previously shown to be involved in a unique transcytosis mechanism for trafficking and sorting neuronal L1/NgCAM to axons (Yap et al., 2008). More recently, Shi et al. (2018) have shown that NSG3 associates with motor proteins to coordinate dynamic microtubule dependent trafficking of LysoTracker^+^ late endosome and lysosome related organelles within axons. Further evidence for NSG2’s unique functions can be inferred by sequence homology and known NSG family protein-protein interactions. NSG1 has been shown to bind to L1/NgCAM, Stx13, GRIP1, and APP (Steiner et al., 2005; Yap et al., 2008; Norstrom et al., 2010), while NSG3 binds clathrin light chain (CLC), the AP1-3 adaptors, and PSD-95 (Xiao et al., 2006; Ha et al., 2012; Muthusamy et al., 2012). Due to the relatively low sequence homology between NSG family members (Muthusamy et al., 2009), it is likely that NSG2 has unique binding partners that can inform the non-overlapping functions of NSG2 and other NSG family members with respect to spatiotemporal divergence in their post-Golgi trafficking.

NSG2 was originally proposed to have a role in secretory trafficking for the following reasons: (1) it is a single-pass transmembrane protein enriched in the Golgi Apparatus and post-Golgi vesicular compartments, (2) its presence in the P3 subcellular fraction of whole-brain ultracentrifugation, and (3) it contains a Secretogranin III-like domain in its C terminus (Sabéran-Djoneidi et al., 1995). Recent evidence from Yap and colleagues confirmed the secretory capacity of NSG2. Blocking endocytosis using a dominant negative Rab5 resulted in accumulation of NSG2 on the surface of hippocampal neurons (Yap et al., 2017). Here, we found that surface GluA2 levels and consequently AMPAR mEPSC amplitude, were significantly increased on NSG2 overexpression, suggestive of a link between its role in the secretory pathway and surface AMPAR levels. It is especially intriguing that NSG2 has an asymmetric synaptic distribution, being present in approximately forty percentage of excitatory synapses. As mentioned, data from this and previous studies show that inhibiting either NSG1 or NSG2 significantly, but not completely, inhibits synaptic transmission. Thus, it will be critical to determine whether other NSG family members occupy the same, or divergent populations of synapses, and whether the effects of their inhibition are additive. Furthermore, understanding their temporal or activity dependence will be critical. For instance, do NSG family members mark specific subsets of synapses, or do NSG proteins transiently visit most or all synapses during development or during particular types of activity? Future investigations to answer these questions should reveal previously unappreciated functional differences between individual excitatory synapses across multiple brain regions.

## Synthesis

Reviewing Editor: Katalin Toth, Universite Laval Decisions are customarily a result of the Reviewing Editor and the peer reviewers coming together and discussing their recommendations until a consensus is reached. When revisions are invited, a fact-based synthesis statement explaining their decision and outlining what is needed to prepare a revision will be listed below.

The current manuscript provides novel information about the functional role of NSG2. The study is technically solid and well designed. However, the reviewers raised few points that should be addressed by the authors. These issues are minor. As for Rev.#2's second point the authors can either tone down the speculation for the potential mechanism or they can support their stated ideas with additional data.

Rev. #1.

This manuscript reports the role of Neuron Specific Gene 2 (NSG2) protein at excitatory synapses. NSG is expressed at development stage and is decreased afterwards. Although the abundance of NSG proteins are very high in neurons, these functions have not been fully studied. It has been reported that NSG2 is highly expressed in Golgi apparatus but further study elucidating the subcellular localization and the functions of NSG2 were missing. In this manuscript, the authors found for the first time that NSG2 is not only expressed in Golgi apparatus but also in the dendritic segments. The authors confirmed the synaptic expression of NSG2 in both human PSCs and mouse primary neurons. In addition, NSG2 and AMPARs were found in the same protein complex in both heterologous cell line and primary neurons. Most importantly, knockout and overexpression of NSG2 reduced the frequency of mEPSC and increased the amplitude of mEPSC, respectively. These results strongly suggest NSG2 is important for the surface AMPAR expression and AMPAR-mediated excitatory synaptic transmission. The overall data quality is high, and the results are convincing. I have only minor comments below.

Minor comments

1) The magnification of Fig. 1A, 1B, 4A, 1-1, 1-2, 1-3 and 4-1 images are too low to derive the author's conclusions. The authors should present the high mag images as the insets.

2) The bar graphs are required in Fig. 1D (% of co-localization between SYN vs NSG2 and HOMER1 vs NSG2) and Fig. 2-1 (% of co-localization between GluA1 and NSG2-mc).

3) The validation of anti-NSG2 antibody is essential in Fig. 4D and 4E.

4) The authors should include sample traces in Fig. 4-2.

5) Line 110: “calcium phosphate method with 10mg plasmid DNA.” I guess 10 mg should be 10 µg.

Rev. #2.

The study presents evidence that NSG2 is a novel AMPAR interacting protein capable or regulating synaptic transmission. ICC localizes NSG2 to somatodendritic compartments, including postsynaptic sites (immunocytochemically defined by HOMER, SYN1, and AMPAR labelling) of cultured neurons. Biochemical evidence supports a direct interaction between NSG2 and AMPAR subunits GluA1 and GluA2 in recombinant systems as well as developing brains. Finally, manipulation of NSG2 levels alters AMPAR-mediated synaptic function (mEPSCs). I have only minor concerns that the authors should address.

1) Were appropriate controls performed to ensure the specificity of the NSG2 gRNAs? In particular did transduction with NGS2 gRNAs alter NGS1 or 3 levels?

2) It is difficult to reconcile the decrease in mEPSC frequency with no changes in AMPAR mEPSC amplitude, PSD95/SYN1/GluA1/GluA2 puncta densities following NSG2 KO. Particularly since NSG2 overexpression increases amplitude but not frequency. The interpretation of decreased PSD-95 signal (ie fluorescence intensity) is tenuous at best and still inadequate to explain decreased frequency. Despite overall similar densities of PSD-95, SYN1, GluA puncta individually in the NSG2 KO have the authors examined the overlap of multiple pre- and postsynaptic signals? For example is PSD95/SYN1 or GluAR/PSD95 colocalization similar in WT and KO neurons?

Minor

1) reference for “Esteves da Silva et al., 2015” on pg 10 is missing from the reference list.

2) The gain or exposure of the NGS2 signal in the image presented in Fig 1B middle panel is dramatically increased relative to that of the other two panels.

3) pg 13 ln 319 “post conception” should be changed to “following birth”.

4)pg 15 lns 377-380. There are no GluA IPs shown in Figure 3A (IPS are for mCherry in 3A) and no GluA IPs presented in the manuscript show Co-IP of NSG2-mC (only unlabeled NSG2 in 3B).

5)pg 17 lns 427-429. The interpretation here and elsewhere that NGS2 KO has a subtle effect on postsynaptic scaffolding based on reduced PSD95 fluorescence intensity is weak. Further, without a concomitant change in GluAR distribution associated with this ‘subtle scaffolding change’ it is difficult to reconcile with reduced sEPSCs. The authors should refrain from such hand-wavy speculation or provide further evidence (eg. EM investigation of PSDs or alterations in PSD95/GluAR colocalization as suggested above).

Extended data provided figures 1-1 to 3-1 largely repeat findings from mouse hippocampla neurons (main text figures) using hPSNs and are not integral to the study. Extended data in figures 4-1 to 4-2 could likely be distilled as additional panels or insets into figures 4 and 5 or simply provided in text.
